# Effects of Energy Parameters on Dimensional Accuracy When Joining Stainless-Steel Powders with Heterogeneous Metal Substrates

**DOI:** 10.3390/ma14020320

**Published:** 2021-01-09

**Authors:** Chunliang Kuo, Yuren Chen, Yupang Nien

**Affiliations:** Department of Mechanical Engineering, National Taiwan University of Science and Technology, #43, Sec.4, Keelung Road, Taipei 106, Taiwan; M10903207@mail.ntust.edu.tw (Y.C.); M10903205@mail.ntust.edu.tw (Y.N.)

**Keywords:** selective laser melting, density, dimension variation, thermal deflection, parametric optimization

## Abstract

This work presents some breakthroughs for obtaining high dimensional accuracy and reliable geometrical tolerance in the joining of stainless-steel powders with heterogeneous substrates. In the laser melting process, the interfacial energy fractions and forces acting at the solid–liquid surface of the melting powders can effectively vary their geometrical shapes and positions before they turn into the liquid phase. When the interfacial free energy is low, the melting powders are near molten, thus the successive volumetric changes can alter the layered geometry and positions. This assumption was validated by a powder-bedding additive manufacturing process to consolidate stainless-steel 316L powders (SLM 316L) on a thin heterogeneous stainless-steel substrate. Experiments were carried out to reveal the effects of the process parameters, such as laser power (100–200 W), exposure duration (50–100 µs) and point distance (35–70 µm) on the resulting material density and porosity and the corresponding dimensional variations. A fractional factorial design of experiment was proposed and the results of which were analyzed statistically using analysis of variances (ANOVA) to identify the influence of each operating factor. High energy densities are required to achieve materials of high density (7.71 g/cm^3^) or low porosity (3.15%), whereas low energy densities are preferable when the objective is dimensional accuracy (0.016 mm). Thermally induced deflections (~0.108 mm) in the heterogeneous metal substrate were analyzed using curvature plots. Thermally induced deformations can be attributed to volumetric energy density, scanning strategy, and the lay-up orientation. The parametric optimizations for increasing in dimensional accuracy (Z_1_: ~0.105 mm), or in material density (~7.71 g/cm^3^) were proven with high conversion rates of 88.2% and 96.4%, respectively, in validation runs.

## 1. Introduction

Appreciable advances have recently been made in the fabrication of metal components for applications requiring strict tolerances and high-load capacity [1]. Success in this area can largely be attributed to the improvements of the powder properties, such as composite structures or engaged nano-particles, in the developed metallic powders to facilitate direct/selective laser melting methods [2,3]. Notable advances have also been made in the development of scanning strategies [4] and the associated integration with finite element method-based numerical models for the in-process control in the melting process [5]. Improvements in the efficiency of these methods has led to success of commercial models capable of meeting the demand for functional parts in large volumes, as well as in the small-batch production of prototypes used to verify fit and assembly [6]. The primary challenge in the further development of this technology lies in the need to achieve high dimensional accuracy, despite the complex interactions among the laser power, the thermodynamic properties of the resulting molten metals, and the scanning parameters.

Table 1 lists the produced dimension variations over multiple laser energy sources when joining metallic powders onto the dissimilar material substrates [7,8,9,10,11,12,13,14]. Aside from the energy event, the inherent properties of the laser-molten metallic powders usually produced defects and reduced material density, which could lead to dimension variations during consolidation. Kruth et al. [3] reported that a decrease in the interfacial energy was consequently in conjunction with the involvement of the increased contaminations of oxide and impurities, which led to alterations of the wetting and adhesion phenomena. As a result, the interfacial free energy was decreased in the laser sintering process, and the resulting dimension variations occurred. In the investigations of printing single lines and layers using water-atomized, gas-atomized and alloyed powders, Rombouts et al. [15] suggested that the use of higher heat conduction in the baseplate probably causes smaller melt volume and leads to the absence of the balling effect; whereas the use of oxygen increases the melt volume but decreases the printed surface quality.

Process optimizations of the laser power density and scanning strategy involving the preferential heat transfer could improve the dimensional accuracy. Kruth et al. [16] investigated the influence of layer thickness (20–80 µm) and laser power (10–200 W) on density, hardness, strength, and stiffness. They reported that a scanning strategy involving the preferential removal of heat could improve the geometric accuracy of small holes (<0.5 mm), the thickness of ribs, and the rounding of sharp corners, while reducing the occurrence of curling and warpage during sintering/melting and heat dissipation. Hong et al. [17] evaluated the effects of the laser power (100–200 W), scan rate (30–270 mm/s), and scan-line spacing (20–200 µm) on the produced surface roughness using selective laser melting of a Co-Cr dental alloy. In the single line formation tests, they found that the balling effect was eliminated under increasing scanning rate and reducing laser power; however, the alloys did not melt completely. In the multilayer formation tests, the moderate scan-line spacing (100 mm/s) produced the smoothest surface (R_a_ ~2 µm).

On the aspect of the thermal-induced defects, Kruth et al. [4] also investigated the temperature gradient mechanism (TGM), which is characterized by thermal deformations and variations between the unconsolidated powder grains and the fully molten units during the mixture of metallic powders. They concluded that a preferable scanning strategy could eliminate the thermal deformation. Li et al. [18] suggested that in the SLM of stainless steel 316L, a constant layer thickness (50 µm) and laser power (100 W), with a low scanning speed (60 mm/s) provides good spreading and consolidation. Increasing the scanning speed (150 mm/s) would result in insufficient heat input and a reduction in the penetration depth of the molten pool, thereby producing incomplete consolidated clusters in successive layers. In contrast, Morgan et al. [19] concluded that a pulse frequency of 40 kHz with laser power of 80 W and scan spacing of 75 µm requires a higher scanning speed (100 mm/s) to achieve a high relative density (~87%). They attributed this to the effects on the melt pool imposed by recoil compression force and a reduction in vaporization pressure caused by the formation of plasma. O’Neill et al. [20] studied a Q-switched Nd: YAG laser with low laser energy (12 W) and high peak power/pulses (60 kHz). They reported that a relatively high scanning speed (250 mm/s) could produce parts of high density with minimal porosity. They attributed this to low consolidation density, which resulted in high recoil pressure leading to the blasting/removal of powder. While the thermal-induced deviations in the layered volume were measured and analysed, the dimensional errors could possibly be identified via observations of the produced density. The density deviations could be controlled via the printing strategy; whilst the dimension errors were resolved via FEA analysis and a computer-added design process. Farzadi et al. [5] reported that a printing time delay of 50–500 ms could have a significant effect on the resulting geometrical features. Spierings et al. [21] commented that the layered component density with regard to the scanning speed could affect the quality of the powder layers and the associated operating parameters, which could significantly change the accuracy of laser melting parts. Vandenbroucke and Kruth [22] optimized the SLM process’ parameters to reduce porosity, resulting in high part densities of 99.8% and 99.9% for Ti-6Al-4V and Co-Cr-Mo alloys, respectively. It was concluded that high dimensional accuracy below 40 micrometers could be obtained, whilst the effects of layer thickness and the produced undulated surface roughness were monitored via parametric control.

When the error compensation was considered from the architecture stage, Paul and Anand [6] proposed that parts could be oriented preferentially to provide a better support structure and thereby minimize regional flatness and volumetric cylindricity deviations. They proposed an algorithm to determine the preferable scanning direction and orientations based on the weightings from a CAD design, instead of using a full support structure. However, they found that the error compensations in the modified CAD model could not satisfy both the contradicting observations of the dimensions’ accuracy and material density simultaneously. The utilization of statistical methods provides a potential avenue for multi-objective optimization. Yadroitsev et al. [23] studied the selective laser melting process for thin wall features using Inox 904L (ANSI 904L) powder, having 95% of particles with a small grain diameter up to 20 micrometers. By forming thin walls by applying a correction factor to the length dimensions, the resultant dimensions of the part in accordance with the specifications given in the CAD model can be achieved. Mukherjee and Ray [24] carried out a systematic review including optimization methods using the fractional factorial design of experiment and response surface methodology (RSM) for observing the parametric effects. This method proved highly effective in identifying the role of operating parameters and suggesting the optimal combinations to achieve specific outcomes.

In this work, the technology of selective laser melting to be investigated was the performance of building mechanical fasteners on a thin substrate (SST 316) by using heterogeneous stainless powder (SST 316L). Aside from the input thermal energy, the interacting interfacial energies are resolved with the thermodynamics of the energy adsorption isotherm. When the input thermal energy transfers the solid powders to liquid, a series of physical phenomena varying with the interfacial energy in the melting process spontaneously occurred from wetting, adhesion, immersion, and spreading, to the formation of melting pools. The influences of the operating parameters (laser power, exposure duration and scanning point distance) on the density and the dimensional variation (diameter and height) were identified via statistical analysis and metrological confirmation. The combinations of the parameters for achieving each of the high material density and dimension accuracy were obtained. Whereas in the bi-objective optimization, the utilization of the response methodology suggesting the preferable combinations of parameters for the best dimension accuracy with no compromise to the material density was detailed.

## 2. Materials and Methods

### 2.1. Thermodynamics of Energy Events

The involved energy events in the laser jointing of composite powders with the heterogeneous stainless-steel substrate via selective laser melting are the input laser energy density and the associated surface energy from wetting to melting. The former would be resolved via the modelling of the heat transfer in the considered volumetric element; whereas the latter might be addressed via a two-component system (solid powder in a liquid melting pool) in a Gibbs adsorption isotherm.

**Figure 1:**
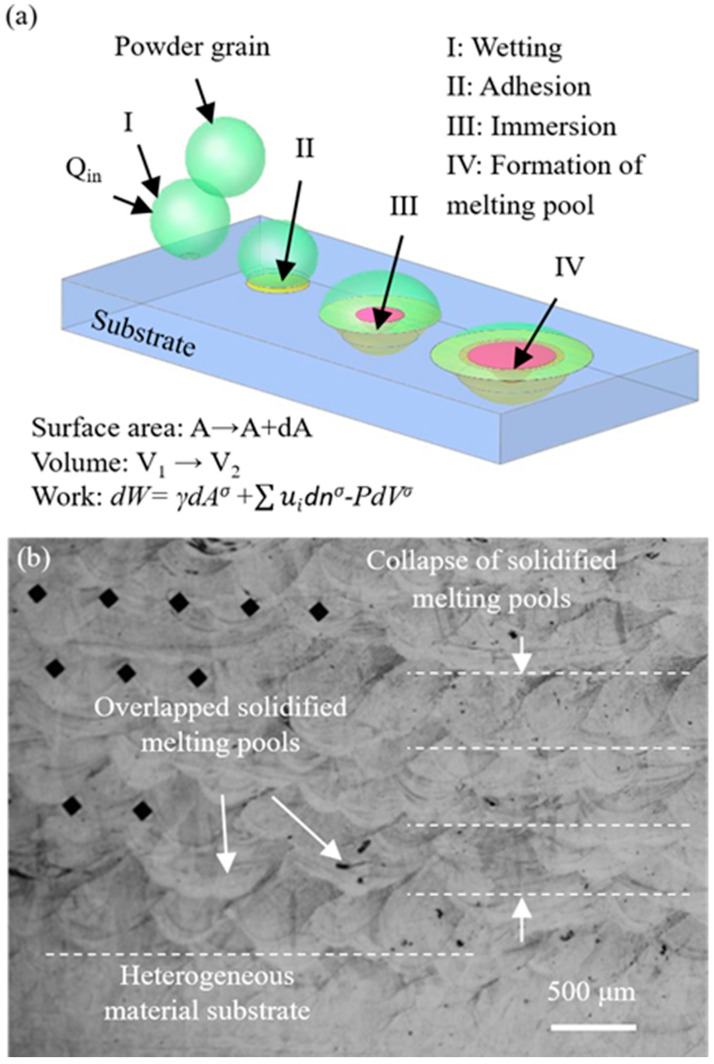
(**a**) Schematic illustration of the consolidation process for a solid grain to the melting pool; and (**b**) observations of the volume changes in solidified melting pools in layers.

Figure 1a depicts the schematic illustration of the progressive process for a solid powder altering its interfacial energy status in consolidation with the liquid phase melting pool. In fact, the melting process could alter the engaged volume and the corresponding surface area; therefore, the work can be resolved by the surface energy and the volume changes. Moreover, Figure 1b presents the recorded observation of the volume changes which reflected the collapses of the melting-pools between layers during solidification. The alterations of the shapes and dimensions in the collapsed melting pools would be measured and characterized.

Aside from the input volumetric thermal energy (*E*), the interacting interfacial energies (*U*) in consideration with the thermodynamics of the energy adsorption isotherm can be expressed as Equation (1):

(1) U=G+T·S−P·V

where G is the Gibbs free energy contribution; *T* is the absolute temperature of the surroundings; and *S* is the entropy of the system. Usually, when the interfacial free energy (*A^σ^∙γ*) increased or the sum of the chemical potential $n_{i}^{\sigma }$ of the i th-component in the phase increases, the Gibbs free energy contribution would increase. Hence, the total energy for the surface phase (*σ*) can be defined from Equation (2).

(2) $$U^{\sigma }=A^{\sigma }\cdot \gamma_{solid-liquid}+\underset{i}{\sum }u_{i}\cdot n_{i}^{\sigma }+T\cdot S^{\sigma }-P\cdot V^{\sigma }$$

Assuming that when the solid phase grain melts into the adjacent liquid melting pool, the temperature and pressure are kept constant in the melting transient. Then, the differentiate equation can be expressed as Equation (3).

(3) $$dU^{\sigma }=A^{\sigma }\cdot d\gamma_{solid-liquid}+dA^{\sigma }\cdot \gamma_{solid-liquid}+\underset{i}{\sum }du_{i}\cdot n_{i}^{\sigma }+\underset{i}{\sum }u_{i}\cdot dn_{i}^{\sigma }+T\cdot dS^{\sigma }-P\cdot dV^{\sigma }$$

when the change of the internal energy $(dU^{\sigma }$) is substituted by the heat input (*dQ*) and the associated work (*dW*) done on the system, the heat input (*dQ*) is reversible under an isothermal process. Then the work done in the consolidations of the solid particle into the melting pool is as expressed in Equation (4). The work done can be divided into three fractions, the work expanding the surface area; the work changing the composition for varying interfacial energy; and the work expanding the volume.

(4) $$dW=\gamma_{solid-liquid}\cdot dA^{\sigma }+\underset{i}{\sum }u_{i}\cdot dn_{i}^{\sigma }+-P\cdot dV^{\sigma }$$

when the input thermal energy transfers the solid powders to liquid, a series of physical activities varying with the interfacial energy in the melting process spontaneously occurs, from wetting, adhesion, immersion, and spreading, to the formation of melting pools. The interfacial energy fractions and forces acting at the solid–liquid surface of the melting powders can effectively vary their geometrical shapes and positions before they turn into a liquid phase.

If the interfacial free energy is low, the melting powders are near molten, thus the successive volumetric changes can alter the layered geometry and positions. As a result, the produced dimension accuracy and geometrical tolerance are degraded. In the analytical studies, the initiated thermodynamics relationships suggest that the input energy varies the surface area together with the work carried out; whilst the entropy of the melting system in a powder grain increases with the surface area. The work carried out in the changing of the surface area in the powder particle can alter the condition of the interface and volume change, and thereby the corresponding physical activities can be resolved. In the experiments, the calculated input thermal energies are allocated controllable parameters, such as laser power, exposure duration and point distance for the observation of the dimensional accuracy and geometrical tolerance.

### 2.2. Experiment Produre and Data Collection

#### 2.2.1. Powder Materials, Laser Source, Scanning Strategy, and Machine Tool Set-Up

**Figure 2:**
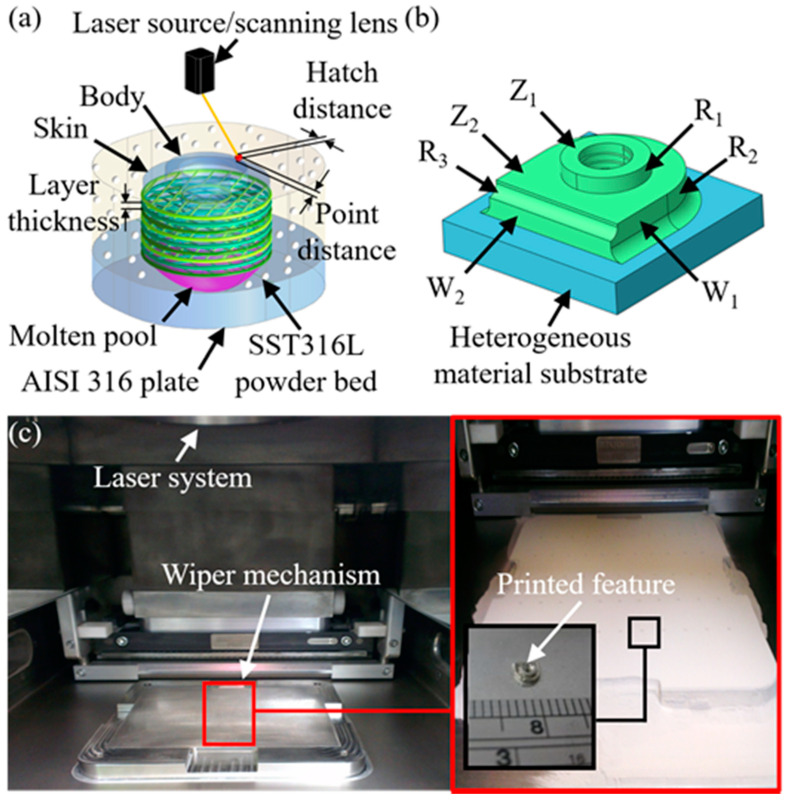
Schematic illustrations of: (**a**) scanning parameters on powder bed; (**b**) observed dimensions in the designed features; (**c**) set-up of the powder-bedding and laser-melting processes.

All the experiments involved the use of stainless steel 316L powder and substrates of AISI 316L for the investigations of the predetermined geometric model. Table 2 presents the chemical composition of the powder and substrate materials. Tests were carried out via a selective laser melting machine (Renishaw AM250, Gloucestershire, UK), which could perform with a high laser power up to 200 W with the lowest focus region of 75 µm on the targeted points. The equipped laser source was utilized with an Ytterbium fiber laser which was characterized with a wavelength of 1070 nm. Figure 2a illustrates the schematic view of the laid-up features of the skin layer and the main body; the defined hatched distance, point distance and the layer thickness in the laser melting powders (SS 316L) on the heterogeneous material substrate (ANSI 316). The scanning strategy involved rotating the orientation 67° clockwise for each layer. Figure 2b presents the post-build, showing the targeted linear and angular dimensions for the evaluations. Figure 2c shows the stage where the prepared substrate before the metal powders are loaded. The particle size of the powders is mainly distributed between 20–40 µm; whilst the substrate was prepared with a 0.8-mm thickness. When the laser melting was undertaken, the build rate was recorded at 5–20 cm^3^/min, increasing with the scanning speed (~2000 mm/s) and the laid-up thickness (20–100 µm). By changing the laid-up orientation and scanning speed, the core body, superficial layer and support structures were established. Layers that function as inter-supports such as the body were fabricated by a high scanning speed of 700 mm/s; whereas layers that function as a skin were created using a relatively low scanning speed of 220 mm/s.

These parameters provide sufficient information to enable the calculation of the scanning length, the defined repetition factor (C) and the volumetric energy density (E).

(5) Scanning volume (V) = w·h·v·t

(6) $$C\;\mathrm{repetition}\;\mathrm{factor}\;=\;\frac{t\;\cdot \;v\;}{s}$$

(7) $$E\;=\;\frac{P}{w\;\cdot \;h\;\cdot \;v\;\cdot \;t}\cdot C$$

where P is the output laser power (W); v is the scanning speed (mm/s); t is the exposure duration (μs); h is the layer thickness (μm); w is the hatch distance (µm); and s is the point distance (μm).

#### 2.2.2. Design of Experiments (DOE)

The typical control variables covering the range generally used for SS 316L were proposed, including laser spot diameter (70–75 µm), layer thickness (30 µm), spacing (125 µm) and number (45) of layers. The parameter values were derived from those previously published in the literature and technical reports (Kruth et al. [4]). Moreover, the energy-related variables, namely the laser power, exposure duration, and scanning point distance, were evaluated on their effects on the part density and produced dimensional variations. The three variables were used as inputs in a Taguchi L_9_ test array, as shown in Table 3, the results of which were examined using analysis of variances (ANOVA) at a 5% confidence level in order to identify the optimal combination of parameters with their percentage contributions (PCRs). In addition, contradicting objectives were integrated into decision-maker equations with assigned weightings for multi-criteria optimizations. Optimum conditions were presented in the response surface plots.

#### 2.2.3. Data Collection

In all tests, the part densities were recorded by the Archimedes method, with a laser sensor (Keyence LK-H025, Osaka, Japan) for recording the level heights of an ethanol bath at a sampling rate of 5 kHz. The measurement of the dimensions and layered geometric features in profile was performed using a laser scanning system (Atos, Braunschweig, Germany). The scanned models were compared to the predesigned CAD model to calculate the mean variations in three measurements for each dimension (Z_1_, Z_2_, R_1_, etc.). The porosity of the material (as measured by volume area) was determined using a Nikon camera-equipped optical microscope with a maximum magnification of 2000×. The captured images presented a resolution of 18 M pixels in a matrix measuring 5184 × 3456 pixels, which resulted in a high resolution of 0.865 µm in the lateral directions. The digital images were processed as micrographs using software (Omnimet, Tokyo, Japan) to conduct the inspections in terms of dimension and geometry measures. Image processing software (Photoshop 14.2.1, San Jose, CA, USA) was adopted to characterize the size and shape of the produced porosity for further analysis.

## 3. Results and Discussion

### 3.1. Material Density

**Figure 3:**
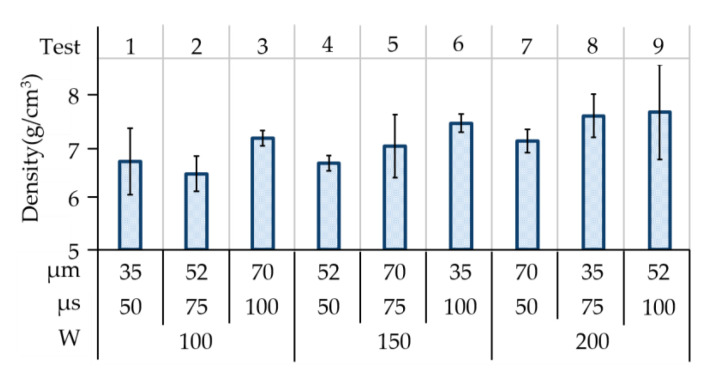
Measurement of density in 3D printed SS 316L features in all (L_9_) tests.

In selective laser melting, the maximum relative density (compared to a solid body) is the primary indicator of the degree of continuity between powder particles. Density tends to vary with the heat applied to the target powders, the interval of exposure, and the scanning strategy. Figure 3 presents the density of the material ranging from 6.50–7.71 g/cm^3^ in all (L_9_) tests. In this study, a high laser power of 200 W, long exposure of 100 µs, and moderate scanning point spacing/distance of 52 µm achieved a maximum average density of 7.71 g/cm^3^, which represented 96.4% of the theoretical material density (SS 316L: 7.99 g/cm^3^) that would be obtained.

**Figure 4:**
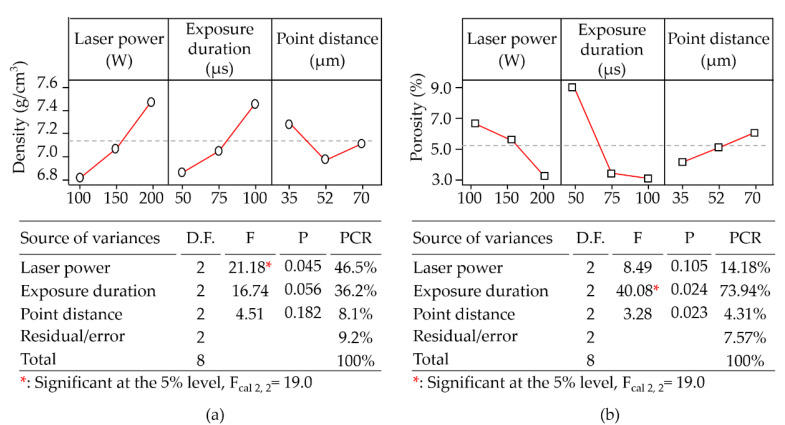
(**a**) main effect plots; (**b**) analysis of variances pertaining to density in all (L_9_) tests.

Figure 4a shows that the main effect plot illustrated that the maximum density was obtained from the highest laser power of 200 W, a longer exposure of 100 µs, and a minimum scanning point distance of 35 µm. As verified by ANOVA, the laser power (F_cal_: 21.18, PCR: 46.47%) and the exposure duration (F_cal_: 16.74, PCR: 36.25%) were greatly influential to the observed material density, despite the laser power being solely beyond the thresholds (F_0.05 2, 2_: 19.0) in the F distribution and presenting statistical significance with regard to density. This is a clear indication that both of these factors are strongly related to density. This is to be expected, due to the fact that the absorption of thermal energy by the powder particles during scanning results in the formation of a melting pool, balanced with the surface energy between the former solidified layer and the ambient surroundings. As a result, the phase transformation occurred continuously along the scanning path, which produced a continuous densification within the melting pool. Providing too much or too little thermal energy could result in boiling or incomplete melting, resulting in the respective formation of irregular or spherical pores. Either defect would result in a low density.

In Figure 4b, the main effect plot revealed that the least porosity would be produced by the operating parameters of a high laser power (200 W), a long exposure (100 µs), and a low scanning point distance (35 µm). Notably, this preferable parameter set with calculated volumetric energy density of 2,031,746.6 W/mm^3^ was higher than that in Test 8 (1,523,810 W/mm^3^) which produced the highest material density in all tests. The received energy was sufficient enough to produce a melting pool where the scanned powder grains transferred into a liquid phase and dissipated heat into the adjacent solidified layers. When the input volumetric energy density was low, the low surface energy and low surface tension dominated the shape and depth in the formation of the melting pool where the layered thickness was in relatively accurate proportions. In contrast, when the input high laser energy overrode the surface energy, the produced melting pools were lobe-shaped with a relatively high depth in penetration to the previous layers; this led to collapses of the melting layers and inconsistent dimension control. In ANOVA, the energy parameter of exposure duration reflected statistical significance with a contribution percentage of 73.94%; whilst the laser power and point distance were less influential to the produced material density. A reduction in material density can be due to the presence of pores or voids in the material. Pores are usually produced by either insufficient heat or overheating; whereas the formation of voids is due to inconsistent shrinkage which opens up cracks, leading to a reduction in the overall density of the material.

**Figure 5:**
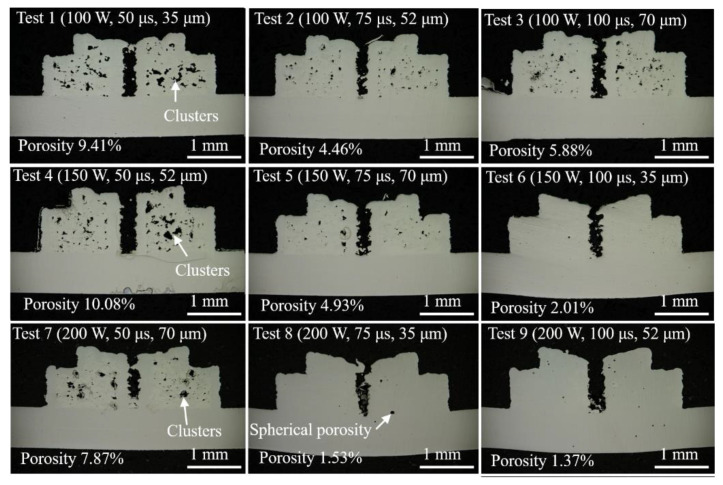
Micrographs showing the porosity of stainless steel 316L workpieces from all (L_9_) tests.

Figure 5 illustrates the types of porosity that can occur in the 3D printing of stainless steel. Test 9 presents porosity (1.37%) lower than that of the other tests. Clearly, the delivery of thermal energy into the powder for a set exposure interval at a given scanning point distance thus initiated wetting, which was followed by spreading or convection among the powder particles, and finally, diffusion. If the supplied energy density exceeded the threshold of the phase transformation, then the solid powders would transform into a liquid in the form of a localized melting pool, thereby reforming the material without the pores during the layering process. This explains why a high laser power (200 W), extended exposure (100 µs), and small scanning point distance (52 µm) are an ideal combination with regard to minimizing pores. Consequently, a lack of heat or an excess of heat would likely lead to problems. The micrograph of the samples in Test 1 shows that the porous material coalesced into stripes, clusters, or large areas with missing material. Layers of loose powder without a well-consolidated base tended to collapse in the form of stripes or clusters, and these effects were exacerbated in cases of low volumetric energy density. Conversely, when the supplied energy exceeded the threshold required to melt the particles, the melting pool tended to vaporize, resulting in the spherical pores shown in the micrographs from Tests 6, 8, and 9.

When comparisons were made between the input volumetric density and the produced density, it was obvious that when the input volumetric energy density was greater than 1,025,641 W/mm^3^, the produced densities delivered appreciable conversion rates of ~95.0% regardless of the parametric combinations. In contrast, when the volumetric energy density was not sufficiently supplied, the scanned region produced pores and coalesced them into clusters, leading to the deterioration of the heat transfer into the successive layers. As a result, the produced densities did not linearly fit into the correlations of the input energy density, but instead fitted with the preferable combination of the parameter sets.

### 3.2. Dimensional Variation

Achieving and maintaining a high degree of dimensional accuracy is particularly challenging when fabricating parts using 3D printing in conjunction with SLM. The task is particularly difficult when using a heterogeneous metal substrate. Inconsistencies can be caused by imperfections induced by mechanical manipulation, inconsistent melting and heat dissipation leading to anisotropic shrinkage, and balling and splashing effects due to vaporization. Mechanical powder-bedding actions can lead to the dislodgment and/or removal of powder in the form of particles or aggregate, with subsequent effects on melting and solidification. The alloying of the powders can produce inconsistent physical changes via the surface tension during melting, and from the wetting, spreading to melting actions. Thus, the non-uniform consolidations of the powders may produce inconsistent dimensions. In contrast, if the input heat for the phase transformation is sufficiently supplied to form melting pools in the consistent sizes, the variations in dimensions between each layer will be able to be controlled.

**Figure 6:**
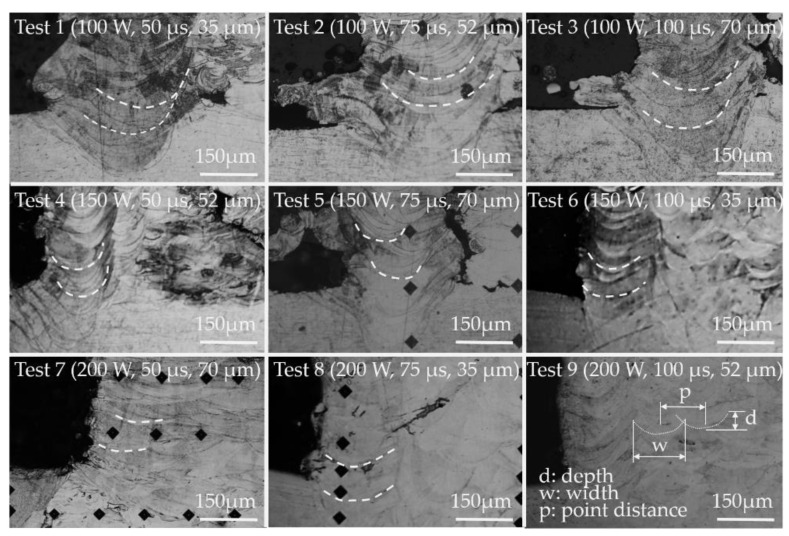
Micrographs showing the lobes of the solidified melting pools at the interface of 316 SLM powders and AISI 316L substrates from all (L_9_) tests.

Figure 6 presents the shapes and penetration depths of the lobed melting pools from the layered 316 SLM powders into the AISI 316L stainless-steel substrate in all (L_9_) tests. When the input volumetric energy density was low, the low surface energy and high surface tension would dominate the shape and depth during the formation of the melting pools, where the layered thickness was controllable and the produced dimensions were relatively accurate. In contrast, when the input high laser energy overrode the surface energy, the produced melting pools were lobe-shaped with a relatively high depth of penetration to the previous layers, which led to collapses of the melting layers and inconsistent dimension control. That is to say, the input volumetric energy could override the effects, while the input heat greatly exceeded the threshold of the melting point of the engaged volume of the layered stainless-steel powders.

**Figure 7:**
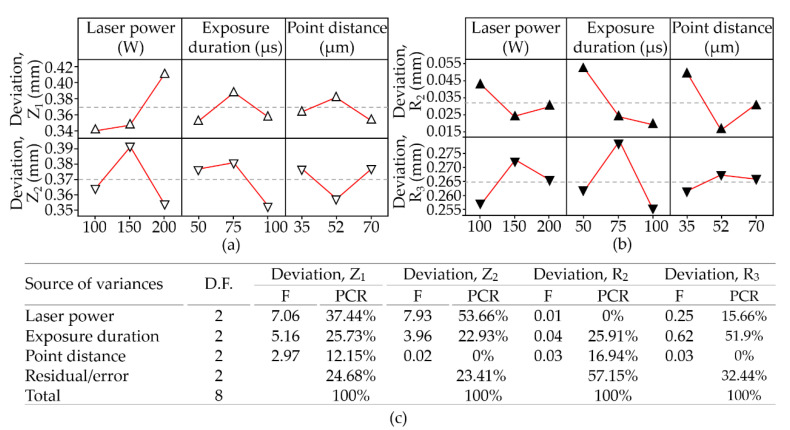
Main effect plots for variations of the (**a**) linear dimensions of Z height; (**b**) radial dimension of R; and (**c**) ANOVA in all (L_9_) tests.

In this work, dimensional variations of interest were in the linear, angular, and radius measurements recorded from the stainless-steel 316L workpieces. It indicated that the Z heights (Z_1_: −0.319 to −0.128; Z_2_: −0.345 to −0.211) were generally lower than the nominal dimensions in the CAD model. It appears that the collapse of the melting pools eroded the support from the previous layers, leading to a recession across the surface. The area near the central hole presented a pronounced depression. The high laser power (200 W) produced a depression of (Z_1_: 0.252 mm; Z_2_: 0.283 mm); whereas a low laser power (100 W) produced a depression of (Z_1_: 0.292 mm; Z_2_: 0.345 mm). In Figure 7, ANOVA indicates that dimension Z_1_ was particularly affected by the power of the laser (F_cal_: 7.06, PCR: 37.44%) and the exposure duration (F_cal_: 5.16, PCR: 25.73%); whereas dimension Z_2_ was affected only by laser power (F_cal_: 7.93, PCR: 53.66%), despite none of the operating parameters being significant to the threshold (F_0.05 2,2_: 19.0) in the F distribution. However, the dimensional accuracy is influenced by the shrinkage and density of the pieces being formed, both of which are nevertheless greatly affected by laser scanning parameters. When these parameters could be controlled and produced a balanced status for the input heat, heat absorption by the powder particles, and heat transfer in the melting pool and the ambience, then the dimension variation could be managed.

It was expected that the main effects plot and ANOVA would indicate a combination of parameters capable of enhancing dimensional accuracy, regardless of particle size and scanning strategy. In fact, this was not the case. The actual powders ranged in size from 25–70 µm, which led to considerable variations in the absorption of heat by particles of different diameters. Differences in heat absorption manifest as variations in the consolidation of particles, ranging from partial melting to full melting.

Table 4 lists the deviations in linear and angular dimensions in the layered SST316L workpieces. It was clear that variations of the regional densities could have a direct influence by the shrinkage rates, and also an indirect influence to the dimensional accuracy. Furthermore, the storage of heat in the powder particles may be influenced by the orientation of the scanning path, wherein additional heat may be transferred from lower layers to upper layers when they happen to overlap. If the excess heat in overlapped areas pushes temperatures far beyond the threshold of melting, vaporization, splashing, balling, and collapse of the melting pool may occur. Similarly, the radial dimensions were examined in terms of diameter (R_1_, radius of a circle), the radius of a semi-circle (R_2_) and an arc (R_3_, radius of a quarter-circle) as listed in Table 4. In Test 9, the relatively small variations: 0.11 mm at R_1_ (−0.072 to +0.038 mm); 0.013 mm at R_2_ (−0.014 to −0.001 mm); and 0.219 mm at R_3_ (−0.251 to −0.032 mm) were simultaneously produced. This might be explained by the fact that improvements in density (<3% porosity) might be reflected in more accurate radial dimensions, despite the fact that ANOVA identified none of the operating parameters as having a significant effect on the test results (R_2_, R_3_). However, anisotropic shrinkage in radii was observed in all tests, regardless of the involved operating parameters.

**Figure 8:**
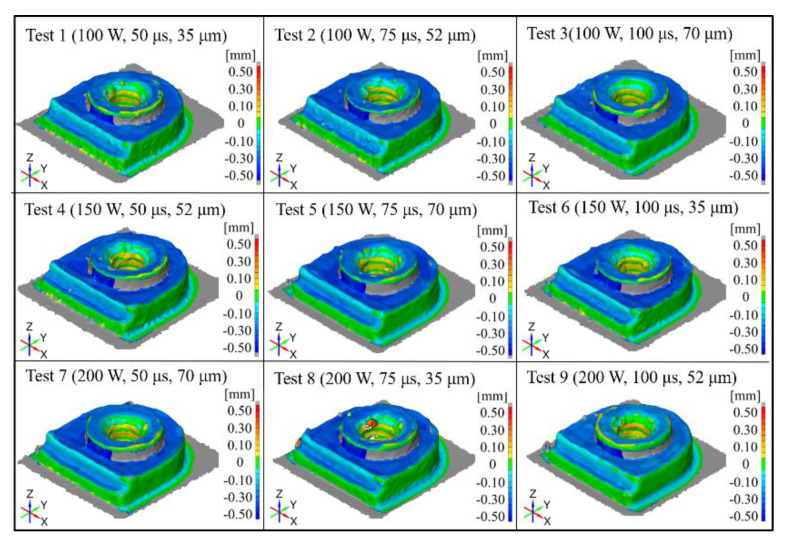
Comparisons between the 3D scanned topography and CAD model for all (L_9_) test array.

Figure 8 presents a three-dimensional topography of the samples in all tests. The R_1_ dimension measured from the curved surface indicates an offset towards the geometrically symmetric center of the constructed features in all tests. This may have been caused by shrinkage, which varied with the amount of heat that was retained in the central area of the features. Similarly, the R_3_ dimensions presented entire offsets towards the geometric center. This is to be expected based on the fact that the maximum offset distance was calculated from the far side to the center, which implied a longer distance over which the effects of shrinkage may be manifested. Surprisingly, the R_2_ dimensions remained between −0.014 mm to 0.094 mm, which is noticeably better than the R_1_ and R_3_ dimensions. It should also be noted that the R_2_ features were constructed layer by layer from the base plane geometrically symmetric to the center, which means that the thermally-induced expansion and shrinkage were somewhat limited. Furthermore, the larger surface area around the circumference from the base plane is no doubt highly effective in the dissipation of heat. The relatively rapid cooling at the circumference could be expected to constrain the geometry to that of the CAD model.

The accuracy of these geometrical constraints was also confirmed in the linear dimensions (W_1_: −0.043 to −0.001 mm) and length (W_2_: −0.443 to 0.017 mm) in all tests. The smallest variation in W_1_ (0.008 mm) was obtained in Test 2 and the smallest variation in W_2_ (0.009 mm) was obtained in Test 3. This implied that accuracy along the X/Y axis was solely influenced by the low laser power (100 W), with relatively low impacts from the exposure duration and scanning point distance. This finding was in-line with the results reported by Kruth et al. [3,16], in which partial sintering led to the reduced latent heat in the particles, compared to full sintering, partial melting, or full melting. This would in turn reduce the thermally-induced errors caused by the melting pool.

### 3.3. Observations of Deflection and Deformation

**Figure 9:**
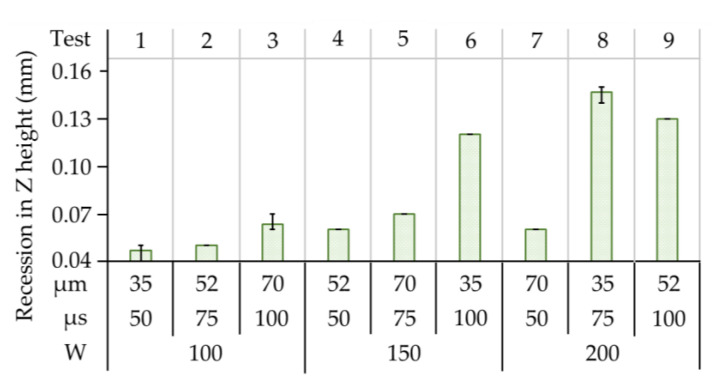
Recession in Z height recorded from stainless steel workpieces in all tests.

The process of selective laser melting generally involves massive heat input to a scanning spot within a very short interval (~100 µs), leading to a rapid increase of temperature localized in the melting pool, with a far lower temperature in the adjacent layers. This phenomenon was clearly observed and confirmed for the scanned regions since the thermal conductivity of the stainless steel was naturally low (16–17 W/m·K). Figure 9 shows the measurement results of the thermally-induced recession in the Z-axis in all tests. The maximum recession values are in-line with that of the input laser energy density in SLM of stainless steel. However, if the calculated input energy density was below the threshold (~770 kW/mm^3^) such as that in Test 1–5 and 7, the deflection values were in the interval of 0.05–0.06 mm, regardless of the operating parameters. In Test 1 (100 W, 50 µs, 35 µm), the energy density was calculated to be as low as 761,905 W/mm^3^ based on a single scan, which resulted in a convex deformation reaching 0.06 mm. In Test 8 (200 W, 75 µs, 35 µm) the volumetric energy density was 1,523,810 W/mm^3^, which increased the amount of deformation by more than 2.2 times larger, up to 0.16mm. This implies that thermal deformation within the material was not influenced solely by volumetric energy density, but also by variations in thermal conductivity, which varied with the density of the molten material. In particular, when the operated laser power, exposure duration, and point distance were exceeding the threshold (~770 kW/mm^3^) for obtaining a full melting phase, the discontinuity of the layered material had an effect on the distribution of heat, producing regional thermal-stress concentration and leading to regional deformation. Nevertheless, the distribution of deformation with respect to displacement in the X and Y-axes was not identical. This difference in temperature resulted in thermal stress between the upper and lower layers, which were out of balance in comparison with the neutral lower layers. This resulted in the formation of a convex surface to release the internal stress. Furthermore, the heterogeneous metal substrate (stainless steel 316) acted as a heat sink, which accelerated the heat transfer. As a result, the massive amount of heat was evacuated via the substrate material, whilst the slow cooling rate in the local melting pool was constrained and finally produced the recession in layers.

**Figure 10:**
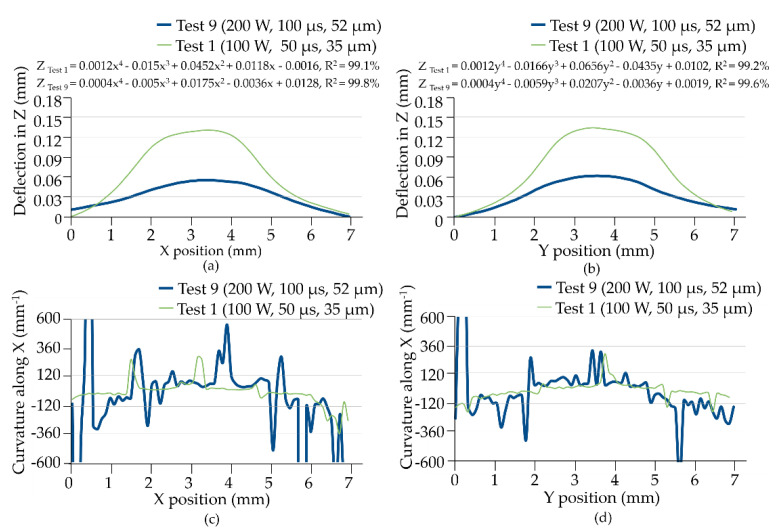
Maximum and minimum deflection values with respect to the (**a**) X position, (**b**) Y position and the corresponding curvatures along (**c**) X position and (**d**) Y position in Tests 1 and 9.

Figure 10a,b present the thermal deformation values in terms of Z height with respect to the associated X and Y positions, respectively. In the stainless-steel workpieces, the deformation was geometrically symmetric to the X and Y-axes despite the fact that the layered features are not geometrically symmetric. In addition, deformation increased with energy density. This explains why the rate at which heat was transferred into the substrates was more or less similar in the X and Y directions, despite the increase in thermal-expansion-induced internal stresses with energy density within the area observed (7 × 7 mm^2^). In Test 9, the greatest deformation in the Y direction was 0.12 mm smaller than the greatest deformation in the X direction, due to the geometrical variation acting as a barrier that caused the dissipation of heat to vary. In the Y direction, the layering of the body along a flat plane (W_1_) in a semi-circle (R_2_) provided a greater area for the dissipation of heat; which reduced the effects of deformation, compared to the W_1_ flat plane in the X direction. These results are in-line with those observed in other areas.

When the applied energy density was low (as in Test 1), the deformation was relatively controlled with little difference between the X and Y directions. In addition, the distribution of heat stored within the observed area (7 × 7 mm^2^) was indicated by the curvature of the deformation along the X and Y-axes, as shown in Figure 10c,d, respectively. In Figure 10c, Test 1 presented an anomaly in the curvature at the interval between 0–2 mm and 5–7 mm. Fluctuations in the slopes from positive to negative are an indication of transitions from straight/flat surfaces to convex surfaces; which implies that thermally induced internal stress was concentrated in the turn points. These also indicated that the corresponding expansions were non-linearly re-distributed in the 0–7 mm interval. Nevertheless, the plots appear relatively symmetrical in both the X and Y-axes. In Test 10, the curvatures in Figure 10c showed that the level interval was evenly distributed, whilst two spikes (±300 mm^−1^) were presented due to sudden changes in curvature in the ramp-up or ramp-down stages. The spikes suggest that the thermal stress was released by means of a radical deformation. Comparison of Figure 10d,c shows that the curvature plot in Test 1 changed from positive to negative in the interval of 6–7 mm in a relatively steady state. This can be attributed to the asymmetric geometry (W_1_, W_2_) of the layered body along the X-axis, which influenced the transfer of heat and associated thermal stress.

### 3.4. Parametric Optimizations of Density and Dimensional Accuracy

Table 5 lists some of the more effective combinations of parameters and the corresponding results obtained in validation tests. No combination of parameters was able to achieve high density as well as high dimensional accuracy. This was due to the contradictions imposed by a high shrinkage rate associated with the consolidation of molten metal, and a low shrinkage rate associated with consolidation after partial sintering. Discontinuities in the physical, thermal, and metallurgical properties of the heterogeneous metal substrate and powders caused localized thermal deflection, which is not generally observed on conventional subtractive fabrication processes. Thermally induced variations were particularly evident at the interface between the printed body and the substrate. Modeling based on multi-criteria optimization was shown to provide statistically reliable results, useful in the minimization of errors.

The application of high energy densities resulted in the formation of materials of relatively high density (Test 6: 1,142,857 W/mm^3^; Test 8: 1,523,810 W/mm^3^; and Test 9: 1,025,641 W/mm^3^) and low porosity (Test 6: 2.01%; Test 8: 1.53%; and Test 9: 1.37%), as shown in Figure 3. However, this approach resulted in the recession of melting pools by thermal shrinkage. Overheating also resulted in vaporization, balling, and splashing effects, which eventually manifest as deviations in the dimensions. Hence, density and dimensional observations could be weighted differently during the process of optimization for specific functions or applications.

## 4. Conclusions

Combining the high laser power of 200 W with the moderate exposure of 100 µs and the low scanning point distance of 52 µm resulted in materials of the highest density of 7.71 g/cm^3^ and a conversion ratio of 96.4% for 316L stainless steel (7.99 g/cm^3^). Materials of low porosity (<3%) were produced when the volumetric energy density was sufficiently high (2,031,746.6 W/mm^3^) to boil and vaporize the powders. The materials of high porosity (>5%) involved the formation of pores in aggregates or clusters, which were produced by the insufficient energy density that was unable to activate the transformation from the solid powder grains into a liquid phase. Imperfections were also caused by variations in the mechanical process used to remove powder from the surface, and variations in shrinkage behavior.

When operating with high laser power and exposure duration, the Z dimension variation was significantly impacted. The recession on the upper layer surface and the induced variations in the Z height evidently occurred. Conversely, in comparison with the linear dimensions (W_1_ and W_2_), the radial dimension variations (R_1_ and R_3_) were slightly influenced by the laser power due to the advantages of the sufficient heat dissipation via a greater circumferential area to the ambient surroundings.

The thermally induced deformation was produced by the process of leveling in which internal thermal stress is relieved through expansion, and occurred in a steady ramp-up/down. However, the spikes suggest that thermal stress was not evenly released due to constraints imposed by geometric features. In the optimization process, the preferable combination for the operating parameters could be suggested by adjusting the weightings of the dimensional accuracy (Z_1_ dimension) and the material density under a balanced level of conversion rates.

## Figures and Tables

**Table 1:** Dimensional accuracy of various layer manufacturing processes [7,8,9,10,11,12,13,14].

Literature Source	Manufacturing Method	Powder Materials	Deviation in X-Axis Direction	Deviation in Y-Axis Direction	Deviation in Z-Axis Direction
Guijun Bi et al. [7]	DLMPD	SS 316L	N/A	N/A	0.12 (mm)
Sadowski et al. [8]	DMLS	IN718	4.2–23.9 (mm)	N/A	N/A
Yu et al. [9]	LENS	SS 316L	0.007–0.101 (mm)	0.39–5.6 (mm)	0.224–0.316 (mm)
Smith et al. [10]	EBM	Ti-6Al-4V	−41.2–2.8 (%)	−40.2–9.12 (%)	1.40–11.3 (%)
Zhang et al. [11]	SLM	Ti-6Al-4V	0.276–0.375 (mm)	0.165 (mm)	N/A
Sun et al. [12]	SLM	Ti-6Al-4V	0.25–2.5 (%)	0–2.25 (%)	−0.8–0.1 (%)
Nguyen et al. [13]	SLM	IN718	−0.04–0.04 (mm)	−0.05–0.05 (mm)	−0.05–0.05 (mm)
Zhang et al. [14]	SLM	SS 316L	7 (%)	7 (%)	N/A
DLMPD: direct laser metallic powder deposition					
DMLS: direct metal laser sintering					
LENS: laser engineered net shaping					
EBM: electron beam melting					
SLM: selective laser melting					

**Table 2:** Chemical compositions of the substrate and powder.

Chemical Element	AISI 316 (Substrate)	SLM 316L (Powder Mixtures)
Carbon	0.08%	≤0.03%
Chromium	10–20%	16–18%
Iron	Balance	Balance
Molybdenum	-	2–3%
Nickel	1–10%	10–14%
Silicon	≤0.075%	0–1%

**Table 3:** Taguchi L_9_ array for SLM of SS 316L.

Test No.	Laser Power (W)	Exposure Duration (µs)	Point Distance (µm)	Calculated Volumetric Energy Density (W/mm^3^)
1	100	50	35	761,905
2	100	75	52	512,821
3	100	100	70	380,952
4	150	50	52	769,231
5	150	75	70	571,429
6	150	100	35	1,142,857
7	200	50	70	761,905
8	200	75	35	1,523,810
9	200	100	52	1,025,641

**Table 4:** Deviations in linear and angular dimensions in SS 316L workpieces.

Test No.	Z_1_ (mm)		Z_2_ (mm)		R_1_ (mm)		R_2_ (mm)		R_3_ (mm)		W_1_ (mm)		W_2_ (mm)	
	Max	Min	Max	Min	Max	Min	Max	Min	Max	Min	Max	Min	Max	Min
1	−0.279	−0.291	−0.254	−0.133	0.055	−0.094	0.094	0.015	−0.063	−0.243	0.020	0.016	0.019	−0.013
2	−0.281	−0.292	−0.267	−0.345	0.063	−0.212	0.010	0.000	−0.062	−0.278	0.013	0.021	0.007	−0.007
3	−0.272	−0.287	−0.230	−0.325	0.045	−0.080	0.026	0.008	−0.059	−0.248	0.026	0.000	0.008	−0.001
4	−0.276	−0.303	−0.271	−0.333	0.030	−0.275	0.026	0.009	−0.062	−0.274	0.039	0.009	0.016	−0.015
5	−0.213	−0.319	−0.271	−0.340	0.027	−0.562	0.015	−0.012	−0.033	−0.284	0.043	0.015	0.017	−0.001
6	−0.278	−0.303	−0.258	−0.316	0.035	−0.301	0.019	0.006	−0.057	−0.263	0.022	0.019	0.024	−0.002
7	−0.270	−0.285	−0.244	−0.321	0.032	−0.423	0.039	−0.002	−0.043	−0.268	0.032	0.039	0.025	0.003
8	−0.128	−0.304	−0.231	−0.326	0.032	−0.023	0.035	0.006	−0.026	−0.279	0.052	0.035	0.022	−0.006
9	−0.192	−0.252	−0.211	−0.283	0.038	−0.072	−0.001	−0.014	−0.032	−0.251	0.030	−0.001	0.034	0.012

**Table 5:** Combinations and validation results.

Observations	Best Combinations			Validated Results		
	Laser Power (W)	Exposure Duration (µs)	Point Distance (µm)	Experimental Results	Theoretical Values	Conversion Rate (%)
Average density (g/cm^3^)	200	100	52	7.710	7.99	96.4
Dimension, Z_1_ (mm)	100	50	70	0.105	0.119	88.2
Dimension, Z_2_ (mm)	100	100	52	0.156	0.184	84.7
Deflection, Z height (mm)	100	75	52	0.098	0.119	82.3

## Data Availability

Data sharing is not applicable to this article.
